# Acute graft-versus-host-disease after liver transplantation: Two case reports and literature review

**DOI:** 10.1097/MD.0000000000044870

**Published:** 2025-10-10

**Authors:** Qiucheng Lei, Xitao Hong, Huazhen Zheng, Lizhuan Su, Huanwei Chen, Feiwen Deng

**Affiliations:** a Organ Transplantation Center, The First People’s Hospital of Foshan (Foshan Hospital Affiliated to Southern University of Science and Technology), School of Medicine, Southern University of Science and Technology, Foshan, Guangdong Province, China; b School of Medicine, Southeast University, Nanjing, Jiangsu Province, China; c Department of Laboratory Medicine, The First People’s Hospital of Foshan (Foshan Hospital Affiliated to Southern University of Science and Technology), School of Medicine, Southern University of Science and Technology, Foshan, Guangdong Province, China.

**Keywords:** case report, corticosteroids, graft-versus-host disease, liver transplantation

## Abstract

**Rationale:**

Acute graft-versus-host disease (GVHD) is a rare but highly fatal complication following liver transplantation (LT). This case report aims to summarize the diagnostic and therapeutic experiences to enhance clinical understanding.

**Patient concerns:**

Two middle-aged male patients underwent LT for acute-on-chronic liver failure and hepatocellular carcinoma, respectively. Both subsequently developed nonspecific symptoms, including rash, fever, and signs of bone marrow suppression.

**Diagnoses:**

The diagnosis of GVHD was confirmed in both patients through skin biopsy and chimerism testing.

**Interventions:**

The primary therapeutic approach involved high-dose corticosteroid therapy and intravenous immunoglobulin.

**Outcomes:**

Despite intervention, the outcomes for both patients were poor. Both patients succumbed to mortality, with causes of death being multiple organ failure and hemorrhagic shock, respectively.

**Lessons:**

GVHD after LT carries a grave prognosis with poor treatment outcomes. This report underscores the critical importance of early diagnosis and intervention for managing this devastating complication.

## 1. Introduction

Graft-versus-host disease (GVHD) is a rare but severe complication of liver transplantation (LT). Incidence rates of this condition have been reported to range between 0.5% and 2%, with mortality rates as high as 85%.^[1–3]^ The pathophysiology involves donor-derived T lymphocytes that mount an immune response against recipient tissues, primarily affecting the skin, gastrointestinal tract, and hematopoietic system.^[3]^ The diagnostic process remains challenging due to nonspecific clinical manifestations, including fever, skin rashes, diarrhea, and cytopenia, which often lead to a delayed diagnosis.^[3]^ Current diagnostic methods rely on clinical evaluation, histopathological examination of skin biopsies and chimerism analysis.

Due to the low incidence of GVHD and its nonspecific clinical manifestations, there is a lack of high-quality, evidence-based medical data. In this study, we present 2 cases of acute GVHD following transplantation, focusing on clinical presentation, diagnosis, treatment, and outcomes. These cases highlight the importance of maintaining a high level of suspicion for GVHD in posttransplant patients presenting with characteristic symptoms. Our findings contribute to the growing body of clinical evidence aimed at improving the diagnostic accuracy and therapeutic outcomes of this devastating complication.

## 2. Case report

### 2.1. Case 1

A 32-year-old man with a 13-year history of chronic hepatitis B presented with jaundice and a poor appetite lasting over 3 weeks. Symptoms of liver failure included severe jaundice, coagulopathy, and hepatic encephalopathy. Total bilirubin peaked at 269 µmol/L, prothrombin time was 48 seconds and international normalized ratio was 3.770. Blood ammonia level was 47.41 µmol/L. The patient was admitted to our hospital after receiving no significant improvement from conservative treatment at multiple other hospitals. A physical examination revealed that the patient was alert, oriented and cooperative, and had severe jaundice and a dark complexion. Examinations of the lungs and heart were unremarkable, and the abdomen showed no tenderness or palpable masses. The patient was diagnosed with acute-on-chronic liver failure, grade II hepatic encephalopathy, and decompensated hepatitis B cirrhosis. His model for end-stage liver disease score was 40 and he was classified as Child-Pugh C. The patient underwent a liver transplant after undergoing a full examination to rule out any contraindications to surgery. We are concerned about a possible intra-abdominal infection. He was treated with carbapenems (1 g IV drip q8h), vancomycin (1 g IV drip q12h), caspofungin (50 mg IV drip qd), and ganciclovir (0.3 g IV drip q12h). Two weeks after surgery, the immunosuppressants were reduced, and intravenous immunoglobulin (20 mg) was added for supportive care. Despite symptomatic management, no significant improvement was observed. On postoperative day 19, the patient was transferred to a higher-level hospital for further treatment. White blood cell (WBC) and platelet (PLT) counts began to decline persistently (see Table 2), and GVHD was diagnosed based on clinical symptoms and skin biopsy results. Despite conservative medical treatment, the patient died 67 days postoperatively due to multiple organ failure.

### 2.2. Case 2

**Figure 1.**
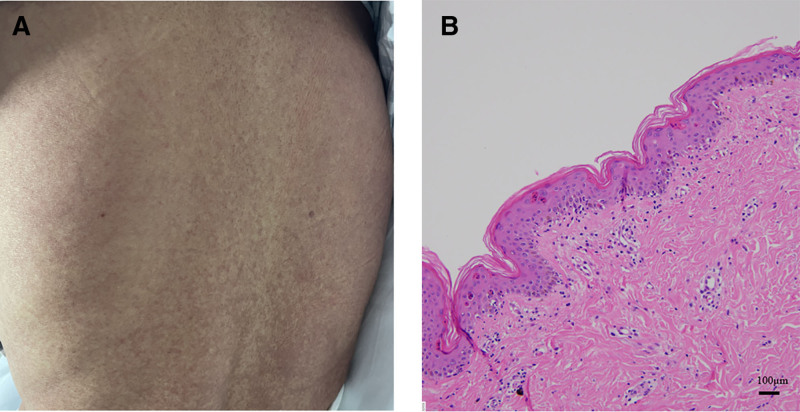
The patient (case 2) with dorsal rash (A) and pathological manifestations on skin biopsy (B) from the left back. Note: (A) Representative photomicrograph of a skin biopsy obtained from the border of a maculopapular lesion on the back, including adjacent unaffected skin. Tissue sections were stained with hematoxylin and eosin (H&E); (B) original magnification: 4 × 10, scale bars: 100 µm).

**Figure 2.**
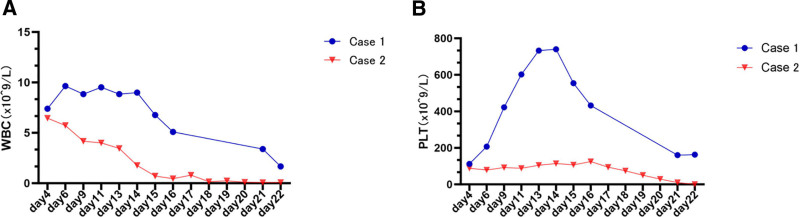
The changes in the levels of leukocyte (A) and platelet (B) in patients after LT. LT = liver transplantation.

A 46-year-old man with a 30-year history of chronic hepatitis B was admitted for comprehensive treatment for liver cancer, which he had undergone for 2 months. Two months ago, he was diagnosed with hepatocellular carcinoma, complicated by a tumor thrombus in the right portal vein (classified as China liver cancer staging [CNLC] Stage IIIA). He subsequently underwent transarterial chemoembolisation combined with targeted therapy (lenvatinib 8 mg orally once daily) and immunotherapy (sintilimab 200 mg intravenous infusion every 21 days). Following successful downstaging therapy, the patient subsequently developed progressive hepatic decompensation, which manifested as abdominal distension, edema of the lower limbs, jaundice, and esophageal variceal bleeding two weeks before admission. On physical examination, the patient appeared alert but anemic and moderately malnourished. He exhibited signs of moderate-to-severe jaundice, manifesting as yellowing of the skin and sclera. A thorough abdominal examination was conducted, which revealed signs of distension, accompanied by a tympanic note and positive shifting dullness. Additionally, mild edema was observed in both the lower extremities. The patient exhibited a model for end-stage liver disease score of 30 and a Child-Pugh class C rating, and consequently underwent orthotopic liver transplantation (LT). Table 1 provides a comprehensive overview of the donor liver. On the fifth day after the operation, the patient exhibited symptoms including bilateral hand tremors and a widespread rash accompanied by itching (see Fig. 1A). These were accompanied by low-grade fever, and there was a further decline in WBC and PLT counts (see Fig. 2). This raised the suspicion of GVHD. A skin biopsy from the affected area on the left side of the back showed incomplete keratinization (see Fig. 1B). Scattered apoptotic keratinocytes were observed in the stratum spinosum, in addition to focal vacuolar changes in the basal layer and lymphocytic infiltration around dermal blood vessels. These findings are consistent with a GVHD skin reaction. The results of the DNA-STR analysis are consistent with a diagnosis of GVHD (see Table 2). The administration of immunosuppressants was ceased, and the patient was treated with methylprednisolone (120 mg) and intravenous immunoglobulin (20 g) for continuous pulse therapy, in addition to recombinant human thrombopoietin, colony-stimulating factors, and platelet transfusions for the purpose of haematological support. Despite the implementation of comprehensive treatment measures for infections, measures to protect the liver, and nutritional support, there was no significant improvement in the patient’s symptoms. By the third postoperative week, the patient’s WBC reached 0.08 × 10^9^/L, and PLT was 0 × 10^9^/L, with concurrent septicemia (blood cultures positive for *Klebsiella pneumoniae* and *Enterobacter cloacae*), and symptoms of hematemesis and melena. The patient ultimately succumbed to multiple organ failure and gastrointestinal bleeding at 22 days postoperatively. A timeline of key clinical events for the 2 patients is presented in Table 3. The present study was approved by the Ethics Committee of First People’s Hospital of Foshan (approval number: Medical Ethics [2025] No. 173).

**Table 1:** The characteristics of the donors.

Patient	Sex	Age	Blood type	BMI	Primary cause of death of donor	Type of donor	Machine perfusion	Age discrepancy between donor and recipient
Case 1	Male*	39	A†	24.2	Extra-heavy craniocerebral trauma	DBCD	NO	NO
Case 2	Male*	44	O†	24.2	Right thalamic hemorrhage into the ventricle	DBD	NO	NO

DBCD = donation after brain death plus cardiac death, DBD = donation after brain death.

* Sex is consistent with the recipient.

† Blood type is consistent with the recipient.

**Table 2:** The chimera test results of T-cell (CD8).

Gene marker	Genetic locus			Proportion of donor-derived cells (%)
	Pre-LT	Post-LT	Liver donor	
	Test sample: fingernail	Test sample: blood	Test sample: liver tissue	
D3S1358	15,15	13,16	13,16	100
D8S1179	14,15	11,12	11,12	100
D2S441	12,14	10,12	10,12	100
D19S433	12,13	13,14	13,14	100
TH01	7,9	7,8	7,8	100
FGA	19,21	24,24	24,24	100
D22S1045	11,18	14,18	14,18	100
D13S317	8,8	9,13	9,13	100
D10S1248	12,15	13,15	13,15	100
D1S1656	13,15	11,13	11,13	100
D12S391	20,23	18,21	18,21	100

LT = liver transplantation.

**Table 3:** A timeline of key clinical events for the 2 patients.

Timeline	Clinical event	Diagnostic findings or treatment
Case 1		
Three wk ago	Poor appetite, abdominal distension, nausea, vomiting, and jaundice	Acute-on-chronic liver failure, chronic hepatitis B virus infection
Day 1	Admission for liver transplant	MELD 40, Child-Pugh C, postoperative rejection management plan: tacrolimus combined with mycophenolate
Day 6	Fever, maximum temperature 37.7°C	Carbapenems (1 g IV drip q8h), vancomycin (1 g IV drip q12h), caspofungin (50 mg IV drip qd) and ganciclovir (0.3 g IV drip q12h)
Day 12	Fever, maximum temperature 38.5°C, WBC: 6.77 × 10^9^/L	Continue original anti-infective regimen
Day 16	Headache, blurred vision, bilateral red eyes	Diagnosed with bilateral conjunctivitis, treated with levofloxacin eye drops
Day 20	Persistent recurrent fever	Transfer to other hospital
Day 25 Day 41	Red rash with leukopenia and thrombocytopenia Persistent fever, low blood pressure	Biopsy-confirmed GVHD Septic shock
Day 68	Multiple organ failure	Death
Case 2		
Two mo ago	Abdominal distension, lower limb edema, accompanied by jaundice	HCC, Bevacizumab 1200 mg IV + Sintilimab 200 mg IV × 2 mo
Day 1	Admission for liver transplant	MELD 14, HCC (CNLC IIIa), postoperative rejection management plan: tacrolimus combined with mycophenolate, corticosteroid reduction protocol
Day 2	Severe thrombocytopenia (platelet count 40 × 10^9^/L)	Platelet transfusions and thrombopoietic therapy
Day 10	Maculopapular rash, tremors of both hands	Biopsy-confirmed GVHD
Day 14	Fever, maximum temperature 38.2°C	Continue original anti-infective regimen
Day 17	Persistent leukopenia, thrombocytopenia, anemia and subtherapeutic tacrolimus levels	Hold tacrolimus; initiate methylprednisolone 2 mg/kg/d IV
Day 18	Basiliximab 20 mg IV on day 18 and 22; Immunoglobulin 20 g IV daily for 10 d	Persistent fever, rash, leukopenia, and thrombocytopenia
Day 23	Uncontrolled gastrointestinal hemorrhage secondary to profound thrombocytopenia	Death

CNLC = China liver cancer staging, GVHD = graft-versus-host disease, MELD = model for end-stage liver disease.

## 3. Discussion

GVHD, first proposed by Billingham in 1966, is a clinical syndrome mediated by healthy T lymphocytes from donor tissues, leading to severe immune reactions and organ dysfunction in the host.^[4]^ It is a complication primarily associated with hematopoietic stem cell transplantation and occurs infrequently in LT, with the first case being reported in 1988 by Burdick.^[5]^ Since 2011, our hospital has performed 291 liver transplants, with only 2 (0.69%) patients developing GVHD. The median time to GVHD posttransplant was 16.5 days.

The extant literature suggests that GVHD is a complex immune-mediated condition primarily driven by donor T lymphocytes that recognize recipient tissues as foreign, leading to severe immune responses and multi-organ dysfunction.^[3]^ The pathophysiology of GVHD is typically categorized into 3 stages: initial damage to the recipient’s immune system, depletion of recipient immune cells by donor lymphocytes, and subsequent targeting of recipient tissues by cytotoxic donor T cells.^[4,5]^ It is noteworthy that the presence of lymphoid tissues in transplanted organs has been observed to influence the likelihood of GVHD development. This is due to these tissues acting as reservoirs for donor T cells.

Several risk factors have been identified that may predispose patients to the development of GVHD, including age discrepancies between donors and recipients, prior immunosuppressive treatments, and the degree of human leukocyte antigen matching.^[6,7]^ A recent study indicated an association between donor infection with human T-lymphotropic virus type I and subsequent development of posttransplant GVHD.^[8]^ The presence of immunocompromised recipients or those with immunologically active grafts has been identified as a risk factor for developing GVHD.^[2,3,6]^ The occurrence of GVHD has been demonstrated to be associated with the dosage of immunosuppressive agents.^[6]^ There is a potential association between pretransplant exposure to immune checkpoint inhibitors and the development of GVHD, particularly in the context of donor-dominant 1-way human leukocyte antigen matching.^[9]^ In the second patient, the recent history of PD-1 immunotherapy may have contributed to an increased risk of GVHD, although such associations are not definitively causal. A recent study posited the hypothesis that the persistence of anti-PD-1 antibodies may activate graft-resident immune cells, thereby triggering GVHD.^[9]^

The diagnosis of GVHD remains challenging and is often dependent on clinical manifestations, including fever, rash, and blood cell count abnormalities. In addition, histopathological confirmation is typically obtained through skin biopsies or the detection of donor T-cell chimerism.^[6]^ Early diagnosis is crucial for effective management of GVHD. Therefore, we recommend that physicians consider this diagnosis in any liver transplant recipient presenting with a maculopapular rash. In the cases under consideration, the timing of onset was consistent with acute GVHD, which occurred within the critical window of 2 to 8 weeks after transplantation.^[6]^ The necessity for precise diagnostic criteria is emphasized by the potential for overlap with other conditions, such as drug reactions or infections, which have the capacity to complicate the clinical picture. In 2022, Cooper et al proposed the utilization of machine learning algorithms, trained on clinical data from patients diagnosed with GVHD, for the prediction of GVHD occurrence during LT procedures.^[10]^ The results demonstrated an area under the curve value ranging from 0.93 to 0.96, signifying the feasibility of leveraging machine learning algorithms to facilitate the diagnosis of GVHD.^[10]^

In the course of the present systematic literature search, the PubMed database was queried using the Mesh term “GVHD” combined with the subheadings “diagnosis” or “therapy.” The search was restricted to publications between January 1, 2014, and December 31, 2024. Subsequent to the screening and quality assessment phases, critical analysis of the included studies suggests that delayed diagnosis and subsequent treatment appear to be significant contributors to the elevated mortality rate in GVHD.^[6,11–14]^ The case reports presented here demonstrate the challenges associated with the diagnosis and treatment of GVHD, and the fatal outcomes observed in patients who developed GVHD after LT despite treatment with steroids and intravenous immunoglobulin. Corticosteroids and intravenous immunoglobulin are the fundamental components of GVHD treatment regimens.^[15,16]^ Patients diagnosed with GVHD should undergo prompt initiation of corticosteroid therapy and should be closely monitored for opportunistic infection. Although the administration of anti-thymocyte globulin has demonstrated efficacy in reducing the incidence of GVHD, it concurrently elevates the risk of life-threatening opportunistic infections. It is important to note that the enhancement of immunosuppression represents a significant challenge in the therapeutic approach.^[17]^ Reducing immunosuppression can potentially rejuvenate the host immune system,^[6,14]^ fostering more effective rejection of alloreactive donor lymphocytes and minimizing infectious complications. In the early stages of GVHD, this strategy should be considered. Nevertheless, it carries the risk of graft rejection and, consequently, has not been widely adopted in clinical practice. The majority of patients succumb to end-organ failure resulting from fulminant sepsis caused by Enterobacteriaceae, vancomycin-resistant enterococci, invasive aspergillosis, or disseminated candida, which are secondary to bone marrow failure, similar to our cases. Table 4 outlines the reported cases, identified risk factors, treatments administered, and their outcomes.^[6,11–22]^ It is worth mentioning that ruxolitinib, a novel Janus kinase 1/2 (JAK) inhibitor,^[23]^ and whole-liver intensity-modulated radiation therapy,^[24]^ are expected to become novel agents and rescue therapies for GVHD after LT, but more robust data are needed. In 2024, Qiu et al reported the first case of using ruxolitinib as an initial therapy for acute GVHD in a liver transplant recipient rather than a salvage therapy.^[23]^ Specifically, ruxolitinib was started 2 days after the onset of GVHD. Subsequently, after treatment, the rash and pancytopenia subsided. Finally, during the 18-month follow-up, the patient had normal liver function and no signs of infection, graft rejection, or immune-related complications. JAK inhibitors show promise in improving allograft survival/function in solid organ transplantation by reducing rejection rates and pro-inflammatory responses.^[25]^ Yet, adverse effects like bone marrow suppression and increased infection risk are major obstacles to their use.^[25]^ Chen et al reported that low-dose whole-liver intensity-modulated radiation therapy could be a rescue therapy for acute GVHD after LT by reducing donor-derived immune cells, and they successfully treated a GVHD patient with this method.^[24]^

**Table 4:** The case reports of acute GVHD after LT.

Reference	Number of cases/total number of LT	The median time to diagnosis (d)	Risk factors	Treatment	Outcome
Zhao et al^[6]^	11/929	16.0	Age disparity > 20 yr, HLA mismatch, HCC, age difference	Immunosuppressant adjustment, steroid, ATG, IVIG, withdrawn or decrement of immunosuppressant	9 dead (sepsis or bleeding) and 2 recovered
Gonultas et al^[11]^	7/2387	59.0	Living donor LT, age disparity > 20 yr, alcoholic liver disease, re-LT	Steroid, ATG, stopping/reducing cyclosporine	5 dead (4 sepsis, 1 bleeding) and 2 recovered
Wang et al^[12]^	1	32.0	Diabetes, HCC, age disparity > 40 yr, viral infections (CMV)	Steroid, ruxolitinib, basiliximab, local liver radiotherapy, antibiotics prophylaxis, plasma exchange, and ATG, stopping tacrolimus and MMF	Recovered
Kaur et al^[13]^	8/1095	36.0	Alcoholic liver disease, HCC, HLA-mismatch	Steroid, ATG, IVIG, and etanercept, stopping tacrolimus and corticosteroid	6 dead (3 sepsis, 2 AHRF, 1 MODS) and 2 recovered
Kang et al^[14]^	6/4294	23.0	HCC, age disparity > 20 yr	Steroid, low-dose calcineurin inhibitor	5 dead (sepsis or bleeding) and 1 recovered
Renganathan et al^[15]^	2/1052	29.0	Age disparity > 20 yr, diabetes	Steroid, IVIG, increase in tacrolimus, G-CSF	Dead (septic shock)
Ofosu et al^[16]^	1	48.0	Age disparity > 20 yr	Steroid, ATG, cyclosporine	Dead (sepsis)
Chesdachai et al^[18]^	12/4585	49.0	NA	Individualized antimicrobial treatment	10 dead (sepsis) and 2 recovered
Elsiesy et al^[19]^	6/604	72.0	Diabetes, HCC, viral infections (CMV), disparity > 20 yr, autoimmune hepatitis, ABO incompatible	Steroid in 4 cases, stopping immunosuppression in 1 case, and no treatment in 1 case	5 dead and 1 recovered
Hung et al^[20]^	1	30.0	Alcoholic liver cirrhosis	TNF-α agent (etanercept), steroid, stopping tacrolimus and corticosteroid	Recovered
Tian et al^[21]^	6/1053	22.2	HCC with age disparity > 20 yr(2), HCC(2)	Immunosuppressant adjustment, steroid, ATG, IVIG/IL-2 antagonists	4 dead (sepsis, multiple organ failure, and cerebral hemorrhage) and 2 recovered
Kim et al^[17]^	2	24.5	Alcohol associated liver disease, HCC, age disparity > 40 yr	Steroid, G-CSF, ruxolitinib, increasing tacrolimus dose	Dead (sepsis, MODS)
Yu et al^[22]^	1	40.0	HCC, age disparity > 20 yr, HLA mismatch	Etanercept	Dead (MODS)

AHRF = acute hypercapnic respiratory failure, ATG = anti-thymocyte globulin, CMV = cytomegalovirus, G-CSF = granulocyte colony-stimulating factor, HCC = hepatocellulare carcinoma, HLA = human leukocyte antigen, IVIG = intravenous immunoglobulin, LT = liver transplantation, MMF = mycophenolate mofetil, MODS = multiple organ dysfunction syndrome, NA = none.

In summary, GVHD is an uncommon yet critically severe complication following LT. Despite the absence of a standardized diagnostic protocol for posttransplant GVHD, thorough diagnostic evaluation and prompt intervention are crucial for enhancing patient outcomes. Emerging therapeutic options such as JAK 1/2 inhibitors and intensity-modulated radiotherapy targeting the entire liver have shown potential as effective alternative treatments for GVHD after LT.

## Author contributions

**Conceptualization:** Huanwei Chen, Feiwen Deng.

**Data curation:** Qiucheng Lei, Xitao Hong, Huazhen Zheng, Lizhuan Su.

**Formal analysis:** Qiucheng Lei, Huazhen Zheng, Lizhuan Su.

**Funding acquisition:** Qiucheng Lei, Feiwen Deng.

**Methodology:** Qiucheng Lei, Xitao Hong.

**Project administration:** Qiucheng Lei, Huanwei Chen, Feiwen Deng.

**Writing – original draft:** Qiucheng Lei, Xitao Hong, Huazhen Zheng.

**Writing – review & editing:** Huanwei Chen, Feiwen Deng.
